# Melioidosis Manifesting as Chronic Femoral Osteomyelitis in Patient from Ghana

**DOI:** 10.3201/eid2801.211800

**Published:** 2022-01

**Authors:** Diana Ayoola Mabayoje, Dervla T.D. Kenna, David A.B. Dance, Caoimhe NicFhogartaigh

**Affiliations:** University College London Hospitals National Health Service Foundation Trust, London, UK (D.A. Mabayoje);; Public Health England, London (D.T.D. Kenna);; University of Oxford, Oxford, UK (D.A.B. Dance);; London School of Hygiene and Tropical Medicine, London, (D.A.B. Dance);; Barts Health National Health Service Trust, London (C. NicFhogartaigh)

**Keywords:** melioidosis, Burkholderia pseudomallei, bacteria, chronic femoral osteomyelitis, osteomyelitis, Ghana

## Abstract

A 33-year-old man from Ghana who had diabetes had chronic osteomyelitis of the femoral shaft develop. Tissue samples from surgical debridement grew *Burkholderia pseudomallei*. He received meropenem, followed by oral trimethoprim/sulfamethoxazole and doxycycline, and fully recovered without complications. Our case report extends the range of countries in Africa as sources of culture-confirmed melioidosis.

Melioidosis is becoming a serious emerging disease worldwide. *Burkholderia pseudomallei*, the causative agent of melioidosis, is a gram-negative, aerobic bacillus found in wet soil and surface water. Human infection occurs by contact with contaminated soil or water through percutaneous inoculation, inhalation, or ingestion. There is often seasonal variation in incidence in association with heavy rainfall. Human cases are mainly reported in high endemicity areas of Southeast Asia and northern Australia, with sporadic reports from other tropical areas, although past research has predicted that many more areas have the prerequisite climate for *B. pseudomallei* (*1*). The purpose of this study was to investigate a case of melioidosis manifesting as chronic femoral osteomyelitis in a patient from Ghana.

## The Study

A 33-year-old man from Ghana who had untreated type 2 diabetes mellitus reported a 2-month history of pain and swelling in the left knee. He had emigrated to the United Kingdom 14 months previously from Ghana, where he had lived in a rural northern area (Bolgatanga Province) for 16 months, working as a building project manager. During this period, he traveled to work on a motorbike over unpaved roads. He had occasional night sweats while in Ghana but had no other symptoms and had not sought treatment. He also traveled to urban cities in Nigeria (Lagos and Abuja) as part of his job. He reported visiting Scandinavia, Germany, Brazil, China, and South Africa over the preceding 7 years, although he had always stayed in urban areas and reported no exposure to soil or surface water.

**Figure 1:**
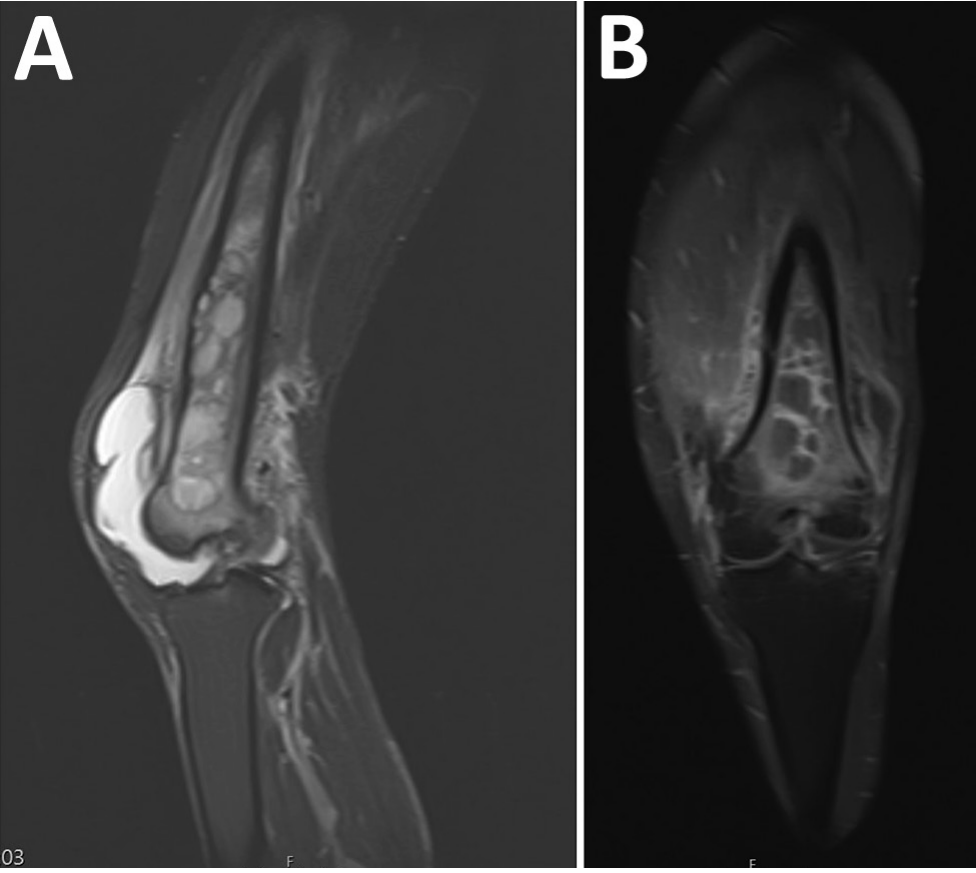
Melioidosis manifesting as chronic femoral osteomyelitis of the left leg in patient from Ghana. A) Magnetic resonance imaging showing extensive femoral osteomyelitis. FFB) Magnetic resonance imaging showing extensive osteomyelitis.

He was febrile (temperature 38.3°C), tachycardic (110 beats/min), and normotensive. His left knee was painful and had a suprapatellar effusion. He had increased levels of inflammatory markers (leukocyte count 11.1 × 10^9^ cells/L, predominantly neutrophils; C-reactive protein level of 221 mg/L; and erythrocyte sedimentation rate of 47 mm/h). Septic arthritis of the left knee was diagnosed. Knee aspirate showed neutrophil polymorphs but a negative Gram stain result and negative cultures. He was empirically given intravenous flucloxacillin, with a plan for a washout. A lytic area was observed on a radiograph of his left femur. Magnetic resonance imaging showed extensive osteomyelitis of the left femoral shaft and metaphysis, including ring enhancement and sinus formation (Figure 1). He underwent incision and debridement of the left femur, during which purulent material was expressed; multiple tissue samples were processed for culture and histologic analysis.

He was empirically given teicoplanin and meropenem. Peripheral blood cultures obtained on the day of hospitalization yielded no growth. Tests results for HIV and syphilis were negative. After 48 hours of culture on standard medium, multiple bone marrow samples yielded an organism identified as *B. pseudomallei* by mass spectrometry. Teicoplanin was stopped, and meropenem started instead.

The isolate was referred to the National Reference Laboratory for Antimicrobial Resistance and Healthcare-Associated Infections (London, UK), which confirmed *B. pseudomallei*. MICs determined by gradient diffusion (bioMérieux, https://www.biomerieux.com; Oxoid, https://www.oxoid.com) and interpreted by using European Committee on Antimicrobial Susceptibility Testing breakpoints (https://www.eucast.org). The isolate was susceptible to meropenem, imipenem, doxycycline, ceftazidime, and trimethoprim/sulfamethoxazole. Multilocus sequence typing showed a novel sequence type, ST1914, a single-locus variant of 3 other *B. pseudomallei* isolates originating in Eritrea, Gabon, and Nigeria (*2*).

**Figure 2:**
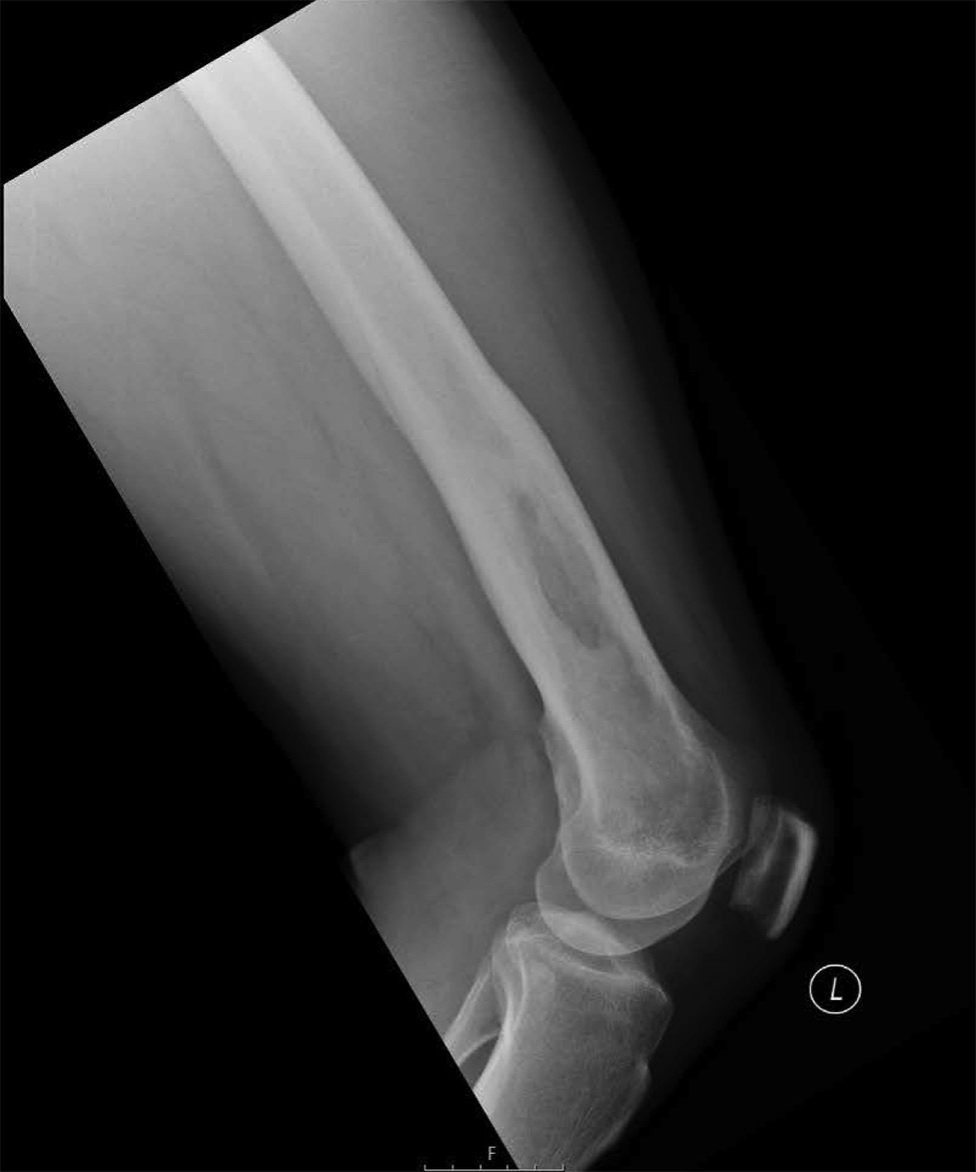
Follow-up radiograph for patient from Ghana with melioidosis manifesting as chronic femoral osteomyelitis of the left leg. Radiograph taken 12 months after initial assessment shows no remaining evidence of infection.

Computed tomography of the chest, abdomen, and pelvis showed no other foci of infection. Because he had labile blood glucose readings and an increased level of hemoglobin A1c, he was given antidiabetic medication. After 14 days of intravenous meropenem, he was switched to oral trimethoprim/sulfamethoxazole (960 mg 2×/d; 160 mg of trimethoprim and 800 mg of sulfamethoxazole) and doxycycline (100 mg 2×/d), completed 2 months of oral antimicrobial drugs, and showed a good clinical response. At the end of treatment, his inflammatory markers had returned to standard levels. Twelve months after he initially sought care, he had full range of movement of his left knee and well-healed surgical scars. He had standard levels of inflammatory markers; a repeat radiograph of his left femur showed changes consistent with his previous debridement and no evidence of ongoing osteomyelitis (Figure 2).

## Conclusions

For our patient, *B. pseudomallei* infection was probably acquired in Ghana. Melioidosis is underreported in known disease-endemic foci, and modeling has suggested that it is probably endemic to 34 countries that have never reported cases, including 24 in Africa; West Africa was identified as the highest risk area, followed by Central Africa on the basis of environmental suitability (*1*,*3*). However, only a handful of sporadic cases have been reported from Africa, probably the result of underdiagnosis caused by resource-limited laboratories and public health systems (*4*–*9*).

Using modeling, Limmathurotsakul et al. estimated an annual incidence of 389 (range 111‒1,446) melioidosis cases in Ghana (*1*). Studies to elucidate the incidence of melioidosis in West Africa, including Ghana, are underway (*3*). Whole-genome sequencing and phylogenomic analysis of *B. pseudomallei* isolates have demonstrated that Australia was an early reservoir, with onward transmission to Southeast Asia and then to southern Asia. Strains from Africa group into a single clade originating from ancestral clades in Asia and human migration from Indonesia to Madagascar >2,000 years ago might have led to dissemination of the organism into Africa with subsequent introduction of *B. pseudomallei* into the Americas through the transatlantic slave trade (*10*).

The timing of this patient’s manifestations and his history of living in rural areas suggest that he was infected in Ghana. Although he had an extensive travel history, including to other known or potential melioidosis-endemic countries, such as Brazil and Nigeria, he stayed in urban settings and had no rural or soil exposure except in Ghana.

Our patient had untreated diabetes mellitus, a major predisposing factor for melioidosis. Increasing prevalence of diabetes mellitus in Africa means an expected corresponding increase in melioidosis cases. In several countries in Asia, hemoglobinopathies are also associated with increased illness and death caused by melioidosis. The milieu of hyposplenism, defective macrophage and neutrophil chemotaxis, and phagocytosis with iron overload are implicated in the pathogenesis of melioidosis in patients who have thalassemia (*11*). A recent case series from the Democratic Republic of the Congo described melioidosis in 3 children who had sickle cell anemia, 2 of whom died (*12*). Hemoglobinopathies are widely prevalent in West and Central Africa and might be an emerging risk factor for melioidosis in settings in Africa.

Approximately 7.6%–14.4% of melioidosis cases have musculoskeletal involvement (*13*–*15*). A longer duration of infection appears to increase the risk for bone and joint involvement. *B. pseudomallei* osteomyelitis requires surgical debridement and prolonged treatment with antimicrobial drugs, usually intravenous ceftazidime or a carbapenem, followed by up to 6 months of high-dose, oral trimethoprim/sulfamethoxazole to achieve cure and prevent recurrent infection, which might occur in <16% of cases after primary infection (*1*). In the case we describe, because of a lack of experience with melioidosis osteomyelitis, the patient received only 2 months of dual oral antimicrobial drugs after 2 weeks of intravenous antimicrobial drugs. Despite this suboptimal treatment, he has had no relapse during 12 months of follow-up. Extensive surgical debridement with good source control might have reduced the risk for recrudescent infection. However, the patient will need to be closely monitored for recurrent infection.

Our case report extends the range of countries in Africa implicated as sources of culture-confirmed melioidosis. We provide additional evidence that melioidosis is underdiagnosed in Africa. This disease should be part of the differential diagnosis of patients with diabetes who have a history of travel in tropical regions and infective symptoms. Strengthening laboratory capacity in Africa will better enable detection of *B. pseudomallei*.
